# Novel Biliary Stent Insertion via Percutaneous Trans-jejunal Approach for Liver Remnant Preservation After Extended Right Hepatectomy

**DOI:** 10.7759/cureus.79925

**Published:** 2025-03-02

**Authors:** Evangelia Florou, Praveen Peddu, Evangelos Prassas, Parthi Srinivasan, Andreas Prachalias

**Affiliations:** 1 Hepato-Pancreato-Biliary Surgery, King's College Hospital, London, GBR; 2 Hepato-Pancreato-Biliary Interventional Radiology, King's College Hospital, London, GBR; 3 Hepato-Pancreato-Biliary Surgery and Liver Transplantation, London Bridge Hospital, London, GBR

**Keywords:** bile leak, biliary anastomosis, biliary fistula, biliary stricture, extended right hepatectomy, hilar cholangiocarcinoma, liver vein deprivation, percutaneous transhepatic cholangiography drainage, portal vein thrombosis, trans-jejunal approach

## Abstract

Extended right hepatectomy (ERH) for hilar cholangiocarcinoma (HCCA) is a complex procedure associated with a high risk of postoperative complications. We present a case of ERH complicated by a biliary fistula at the hepatico-jejunostomy site, which led to a severe anastomotic stricture. Traditional percutaneous transhepatic drainage repeatedly failed in this case. The stricture was ultimately treated with trans-jejunal metal stent insertion, successfully restoring bilio-enteric drainage.

A 62-year-old male patient was diagnosed with HCCA. After staging, he underwent liver vein deprivation to augment the future liver remnant (FLR), followed by curative resection via ERH (segments I and IV-VIII). Postoperatively, a bile leak at the anastomotic site resulted in a bilio-cutaneous fistula. While conservative management led to a gradual resolution, the fistula caused a tight anastomotic stricture, leading to obstructive jaundice. Traditional percutaneous transhepatic drainage attempts repeatedly failed to traverse the lengthy stricture. Consequently, a novel procedure of inserting a metal biliary stent via a percutaneous puncture of the jejunal loop was attempted.

Under CT guidance, the Roux loop was catheterized and distended with contrast, and a guidewire was positioned inside. The patient was then transferred to the angiography suite. Under fluoroscopy, a transjugular intrahepatic portosystemic shunt needle punctured the bile duct stump, providing access to the Roux loop. A fully covered metal stent was successfully deployed crossing the anastomosis re-establishing bilio-enteric drainage. This novel radiological intervention salvaged the remnant liver when standard approaches had failed.

Hepatico-jejunostomy stricture following ERH is a critical postoperative complication that can severely compromise FLR function. Surgical options in such cases are limited and pose significant risks. Radiological intervention offers a promising alternative, enabling effective drainage even in the most challenging postoperative scenarios.

## Introduction

Hilar cholangiocarcinoma (HCCA) accounts for 50-60% of all cholangiocarcinomas, but only 20% of cases are eligible for surgical resection with curative intent [1,2]. Upfront surgery remains the mainstay of treatment, and extended liver resection is often necessary to achieve negative margins when lymph nodes are not involved. The five-year survival rate for this group of patients approaches 40% [1,2].

Future liver remnant (FLR) augmentation is often required to minimize the risk of post-hepatectomy liver failure (PHLF) [2]. Extended right hepatectomy (ERH) carries significant risks, including PHLF (5-58%), sepsis (11%), and bile leakage (5-11%), which are among the leading causes of mortality (8%) [3]. This procedure is best performed in high-volume expert centers with access to specialized surgical, radiological, endoscopic, and intensive care expertise [1,3].

## Case presentation

A 62-year-old Caucasian male presented with obstructive jaundice. Investigations identified a hilar stricture consistent with HCCA. Staging did not reveal distant metastases, and biliary drainage was achieved via bilateral biliary stent insertion through endoscopic retrograde cholangio-pancreatography. Liver vein deprivation (LVD) was performed for FLR augmentation before resection. This procedure included percutaneous embolization of the right portal vein and liver segment IV branches and transjugular access occlusion of the middle and right hepatic veins using an Amplatzer plug.

**Figure 1:**
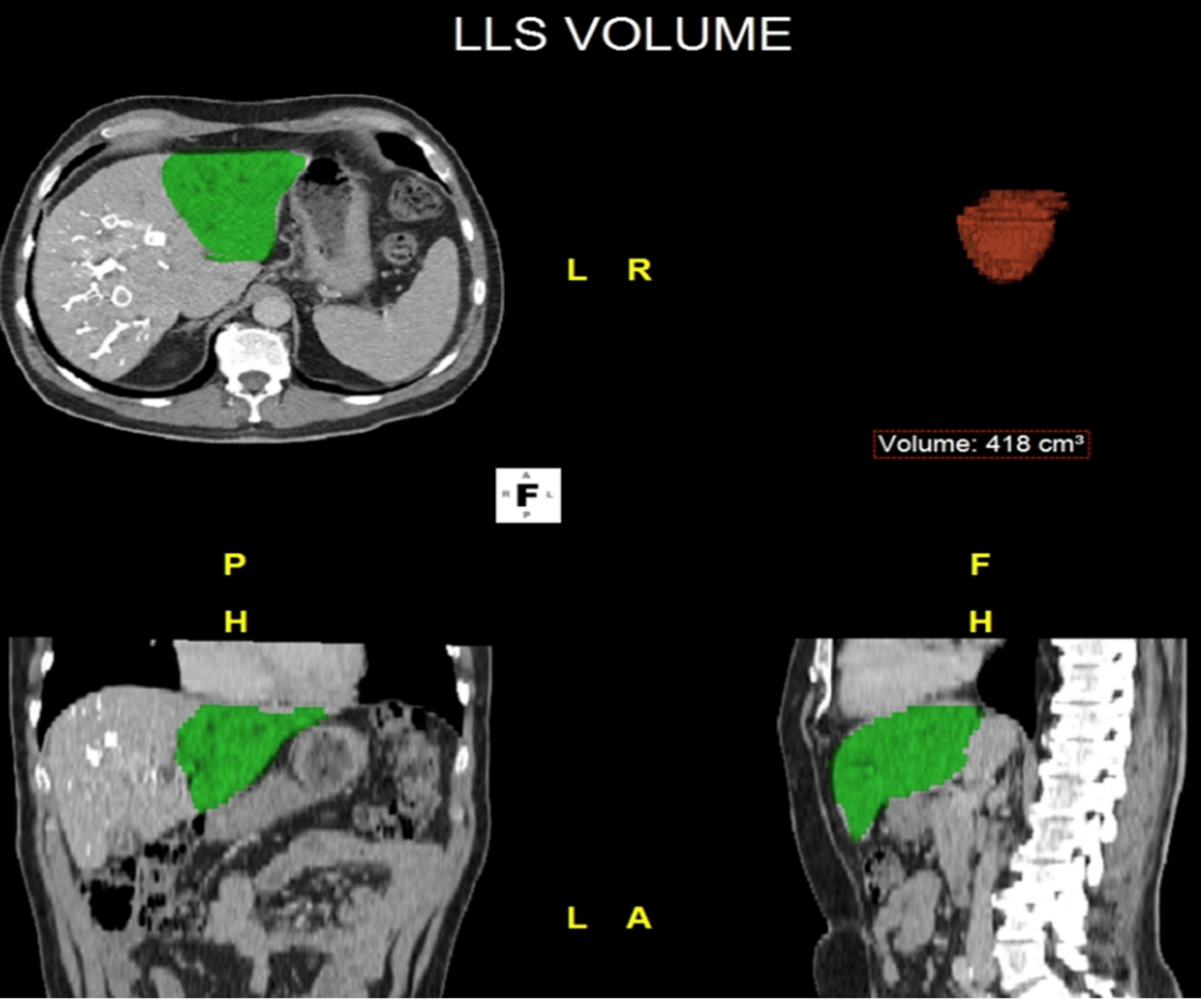
CT-derived volumetry of FLR post LVD Based on CT imaging, volumetric analysis calculates FLR after volume manipulation prior to extended liver resection. In this case, LVD was performed in which the right portal vein as well as the right hepatic veins are occluded to provoke contralateral liver regeneration. The final volume calculation of FLR is 418 cm³. CT: computed tomography, FLR: future liver remnant, LVD: liver vein deprivation

**Figure 2:**
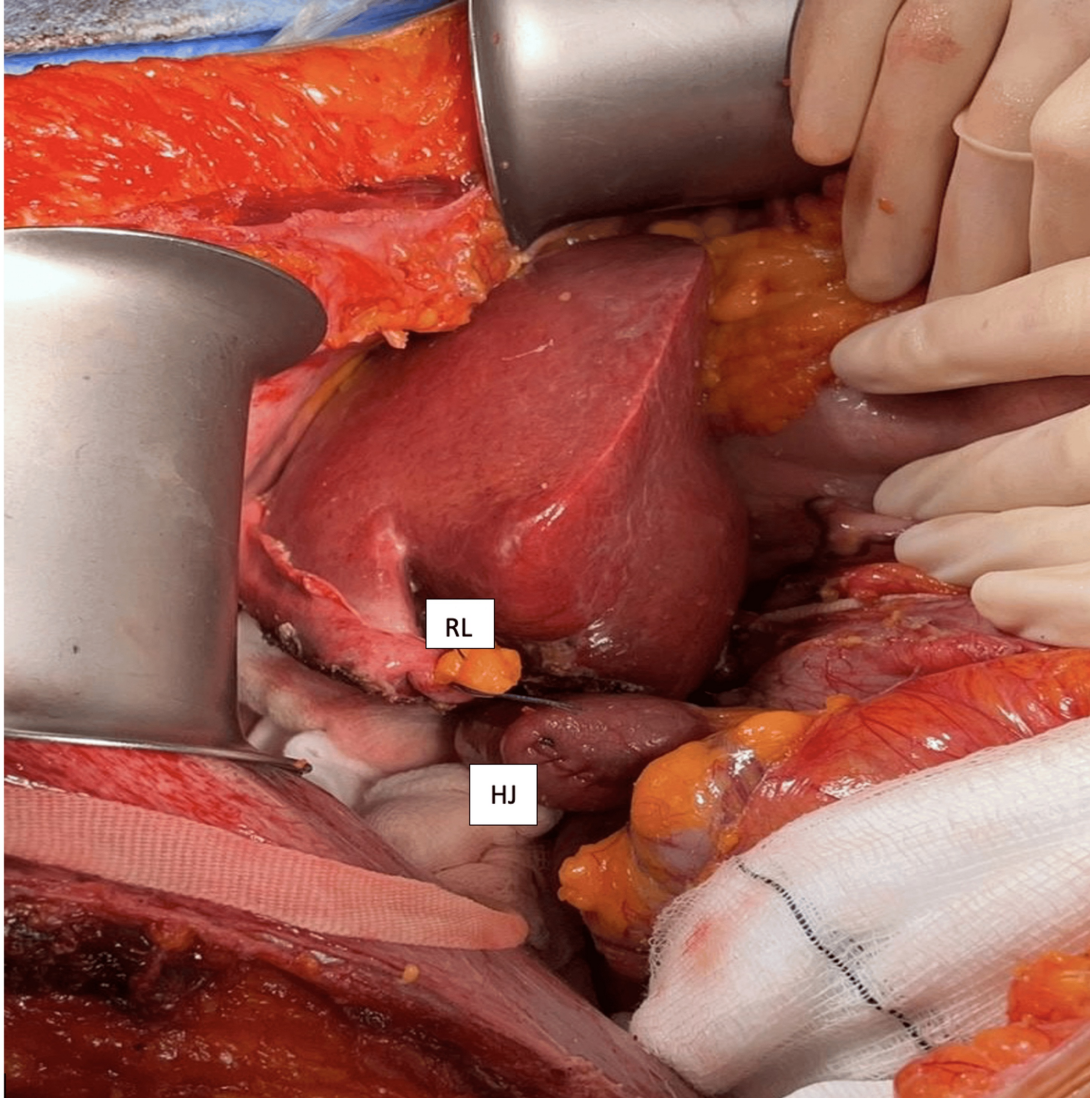
Intraoperative image ERH (segments I and IV-VIII) with Roux-en-Y hepatico-jejunostomy. The RL was marked to demonstrate the level of parenchyma resection. The regenerated left lateral segment represents the future liver remnant. The small bowel stump carrying the HJ. RL: round ligament, HJ: hepatico-jejunostomy, ERH: extended right hepatectomy

CT imaging four weeks post-LVD confirmed satisfactory augmentation of the left lateral segment, with an FLR/BW ratio of 0.58, which was deemed sufficient for subsequent resection (Figure 1) [4]. An ERH (segments I and IV-VIII) was performed, along with extrahepatic biliary tree excision and bilio-enteric Roux-en-Y reconstruction (Figure 2). Histological examination of the surgical specimen confirmed peri-HCCA, with resection margins free of tumor (pT2N2R0).

The procedure was uneventful, and the patient returned to the ward after a 12-hour stay in the high-dependency unit. During the early postoperative period, the patient developed signs of mild PHLF, indicated by the presence of ascites and a mildly elevated bilirubin level, which peaked at 60 µmol/L, thus meeting the 50:50 criteria for potential high postoperative liver failure [5].

**Figure 3:**
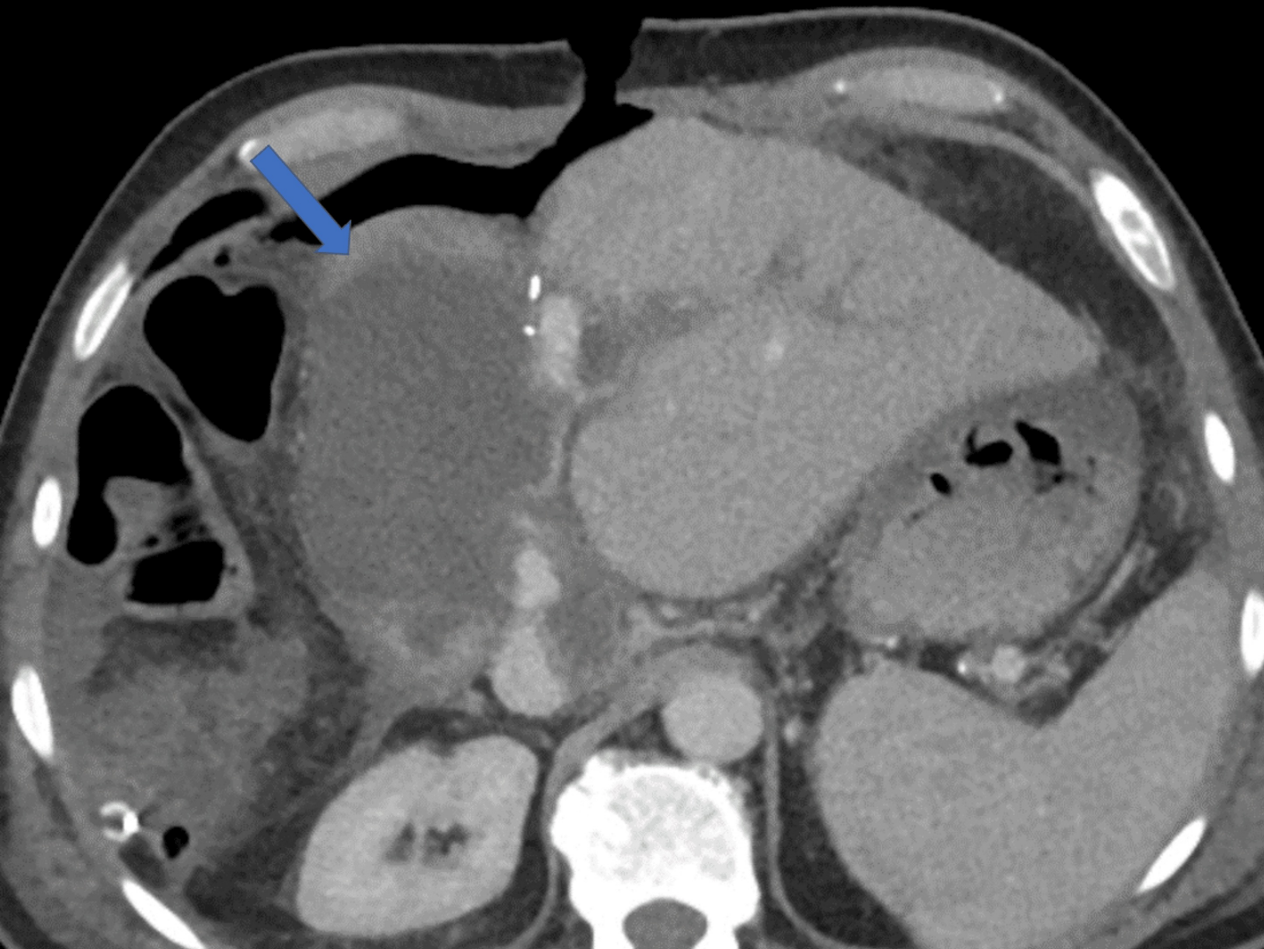
Postoperative CT imaging A CT scan demonstrates a collection (arrow) compressing the hepatico-jejunostomy, resulting in a bilio-cutaneous fistula. The collection appears not amenable for drainage, as it is lying under the small bowel loop carrying the hepatico-jejunostomy. CT: computed tomography

**Figure 4:**
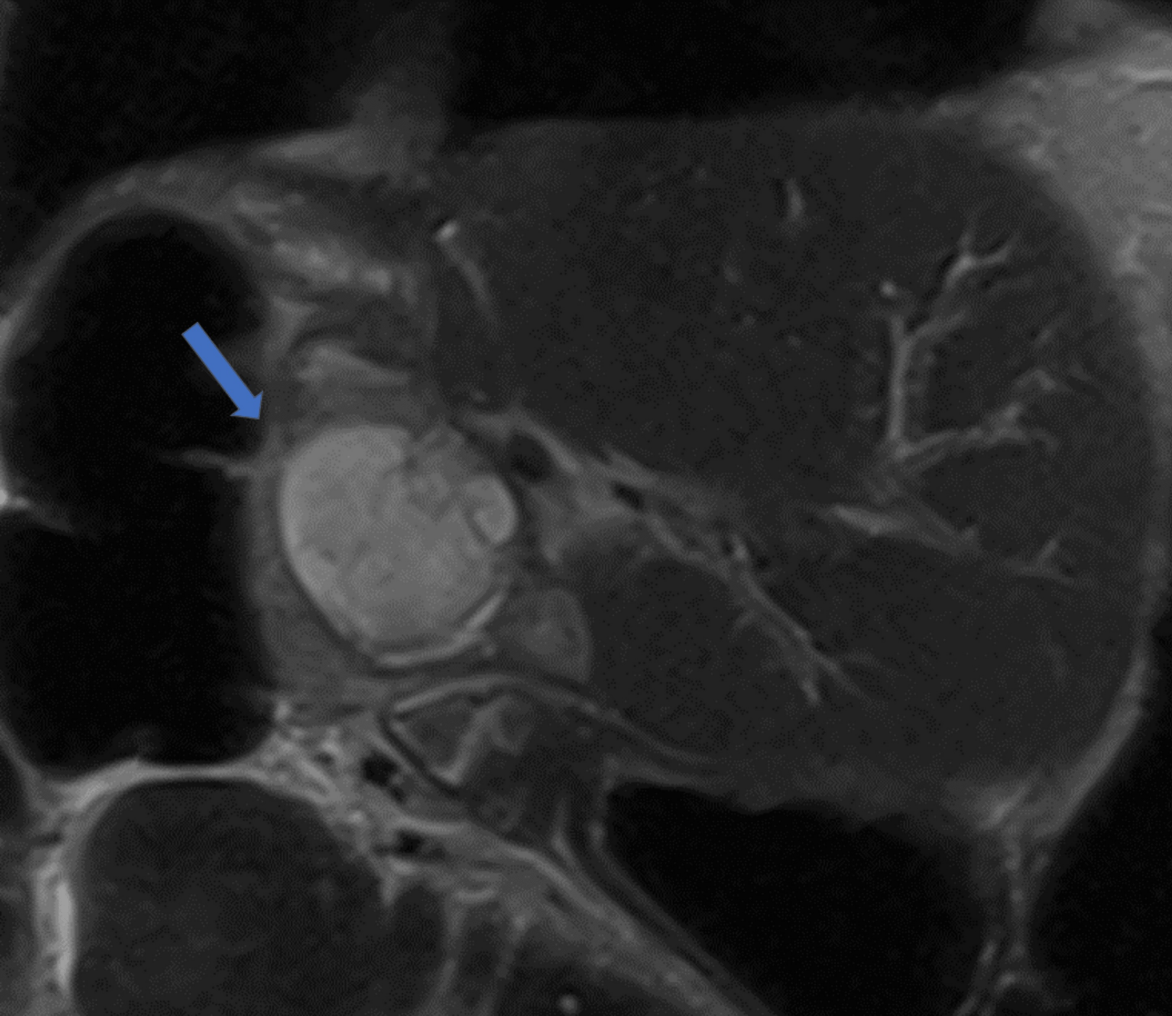
Postoperative MRI imaging MRI coronal view demonstrating the complex collection (arrow) causing pressure to the overlying hepatico-jejunostomy. MRI: magnetic resonance imaging

By the end of the first postoperative week, in addition to the observed PHLF, a bile leak of 50 mL per day was noted, originating from the middle portion of the incision. CT imaging revealed a collection compressing the hepatico-jejunostomy, which was deemed unsuitable for radiological drainage due to its position (Figure 3). Further assessment with an MRI scan confirmed the absence of a safe window for radiological drainage (Figure 4).

While a bilio-cutaneous fistula was established, producing an output of 200 mL per day, a repeat CT scan revealed mild intrahepatic biliary tree dilatation and increased collection size. An attempt at percutaneous drainage was unsuccessful, as no safe radiological window was available to access the collection without risking bowel perforation. The bilirubin level continued to rise, reaching 200 µmol/L by the end of the second postoperative week.

During this period, management included broad-spectrum antibiotics and antifungals, effectively treating recurrent sepsis episodes. Nutritional support was provided through complementary total parenteral nutrition and oral intake.

One month after surgery, a repeat CT scan and magnetic resonance cholangio-pancreatography were performed to further assess the anastomotic site. Imaging showed a reduced collection size and the development of a stricture at the anastomotic site. A percutaneous cholangiogram (PTC) was performed, and an external drain (Navarre Universal Drainage Catheter, BD, UK) was successfully inserted, alleviating the jaundice, which had plateaued at 220 µmol/L.

**Figure 5:**
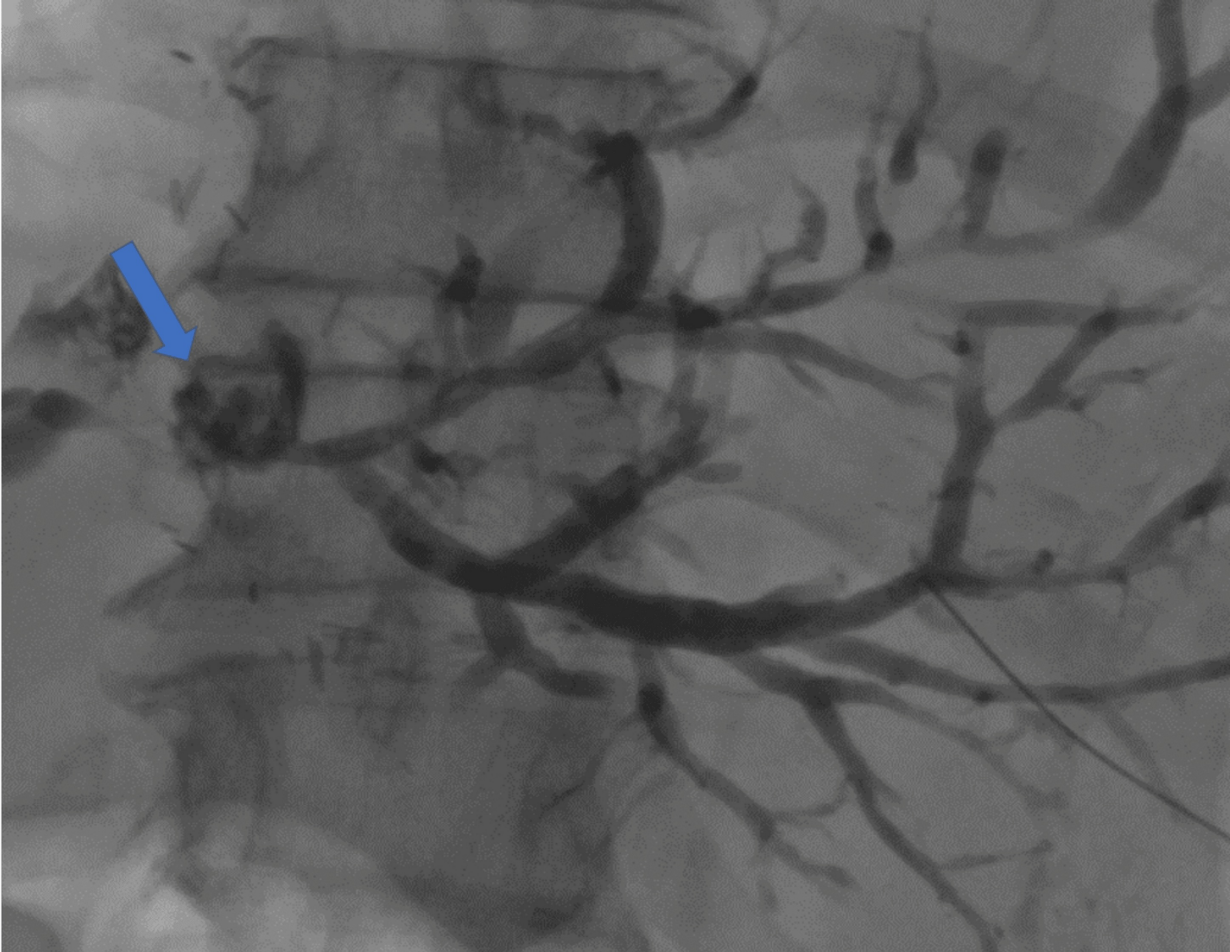
PTC imaging PTC demonstrating a dilated biliary tree with no contrast enhancement of the bowel, signifying a stricture at the anastomotic site (arrow). PTC: percutaneous transhepatic cholangiogram

Following PTC and drainage, liver function tests improved, bilirubin levels normalized, and the bilio-cutaneous fistula gradually resolved. An attempt to internalize the PTC was made two months postoperatively. However, due to the tight and lengthy stricture, internalization failed three consecutive attempts over one month (Figure 5).

Due to the substantial risks associated with revision surgery, a radiological trans-jejunal approach was deemed the most prudent course of action. The radiological intervention is described below. The initial approach was once again via the percutaneous transhepatic route. However, after multiple attempts to cross the stricture via this approach, it became evident that it was extremely tight and lengthy. Thus, anastomotic dehiscence was suspected, with the collapsed Roux loop appearing further from the anastomotic site. It is possible that the initial hematoma led to anastomotic dehiscence, as attempts to traverse the stricture resulted only in the extravasation of contrast from the left bile duct.

**Figure 6:**
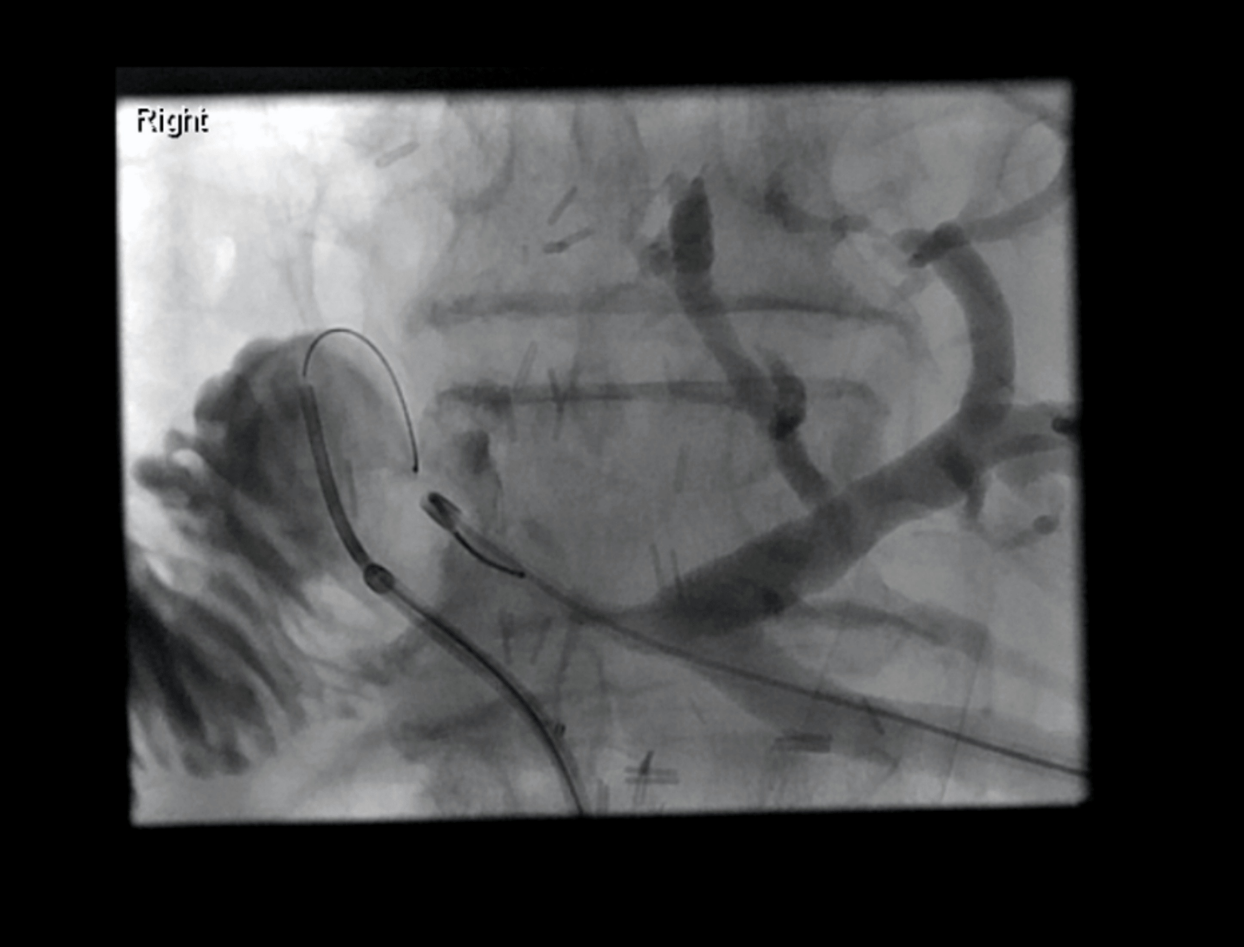
Novel biliary stent insertion via percutaneous trans-jejunal approach The Roux loop is catheterized, and a sheath is in place. The loop is filled with contrast, and a cholangiogram via the PTC is performed to outline the biliary system. PTC: percutaneous transhepatic cholangiography

**Figure 7:**
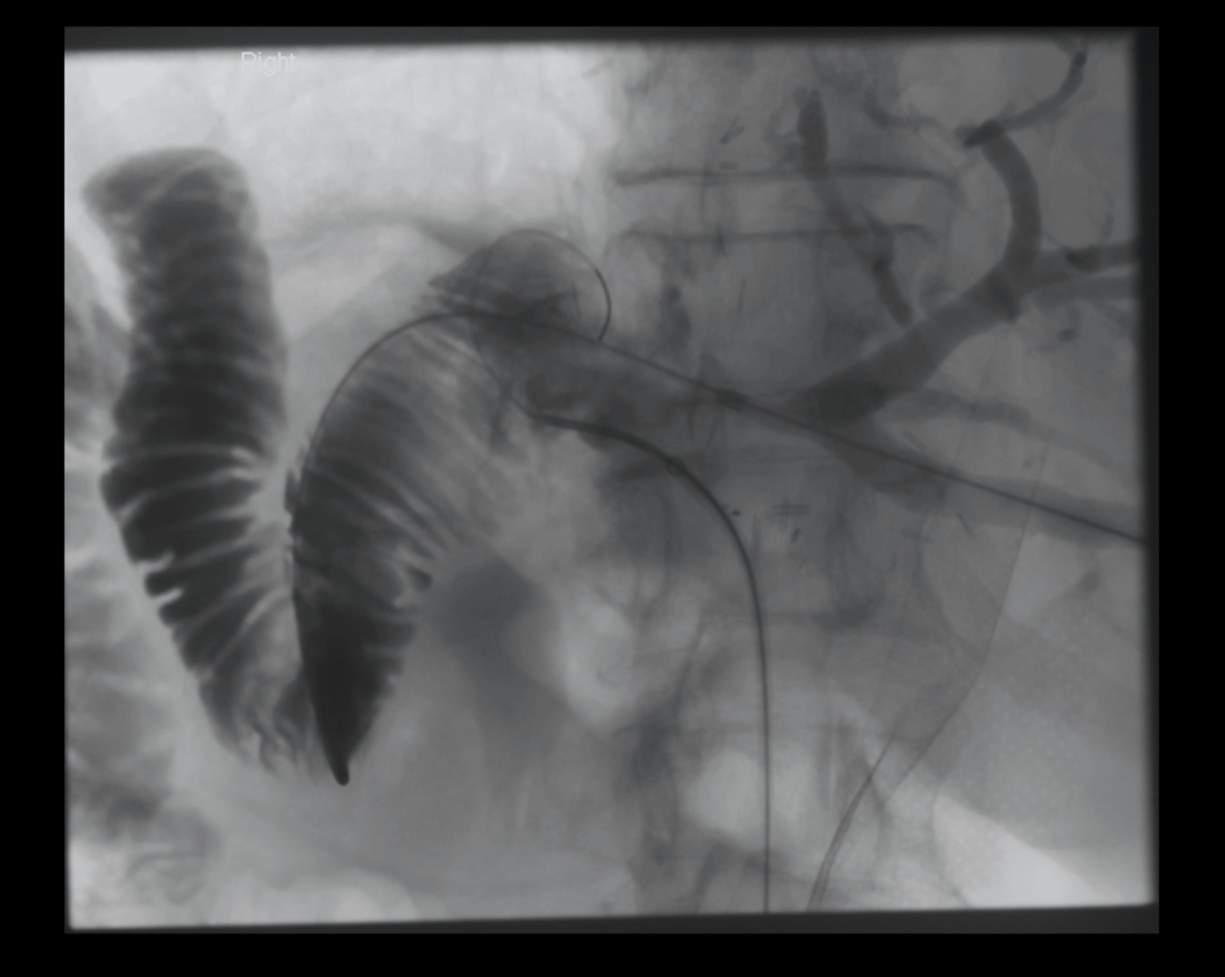
Novel biliary stent insertion via trans-jejunal percutaneous approach The Roux loop was further distended to enable close proximity to the cut surface of the liver at the level of the anastomosis.

A hybrid interventional procedure was planned. Under CT guidance, the collapsed Roux loop was catheterized using the Seldinger technique and distended with air and contrast. A 6 French sheath (BRITE TIP Sheath, Cordis, USA) was inserted into the Roux loop, and the patient was then transferred to the angiography suite. During angiography, a cholangiogram was performed through a sheath in the left duct to delineate the biliary system (Figure 6). Simultaneously, the Roux loop was further distended to bring it closer to the cut surface of the liver at the level of the anastomosis (Figure 7).

**Figure 8:**
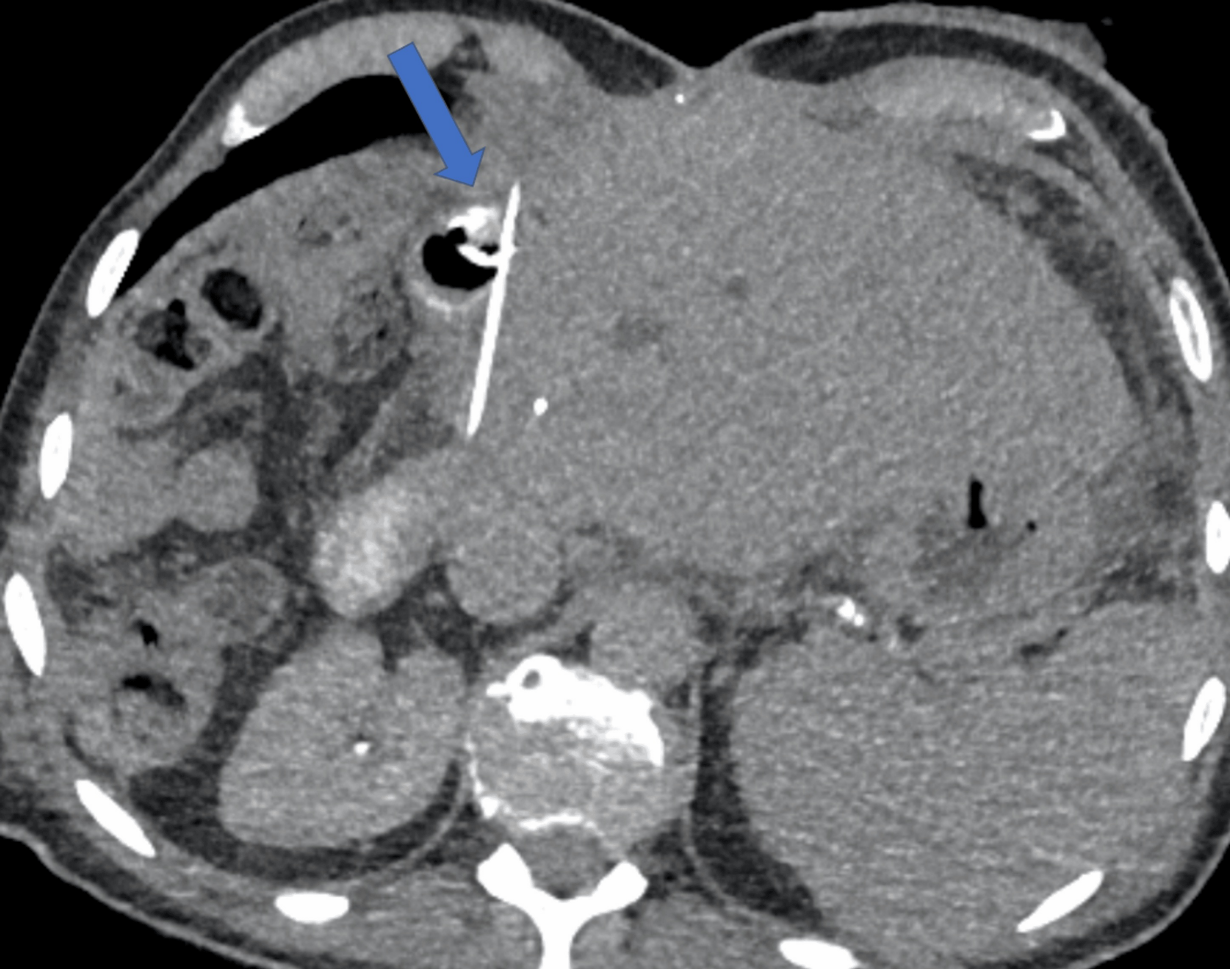
Novel biliary stent insertion via percutaneous trans-jejunal approach Roux loop catheterization under CT guidance using a TIPS needle (arrow). CT: computed tomography, TIPS: transjugular intrahepatic portosystemic shunt

A guidewire (Storq 0.35, Cordis, USA) was placed in the Roux loop. Using this as a target under fluoroscopy, the Roux loop was punctured from the bile duct stump with a transjugular intrahepatic portosystemic shunt (TIPS) needle (Rosch-Uchida trocar stylet inside a 5.2F catheter, Cook Medical, USA) (Figure 8).

**Figure 9:**
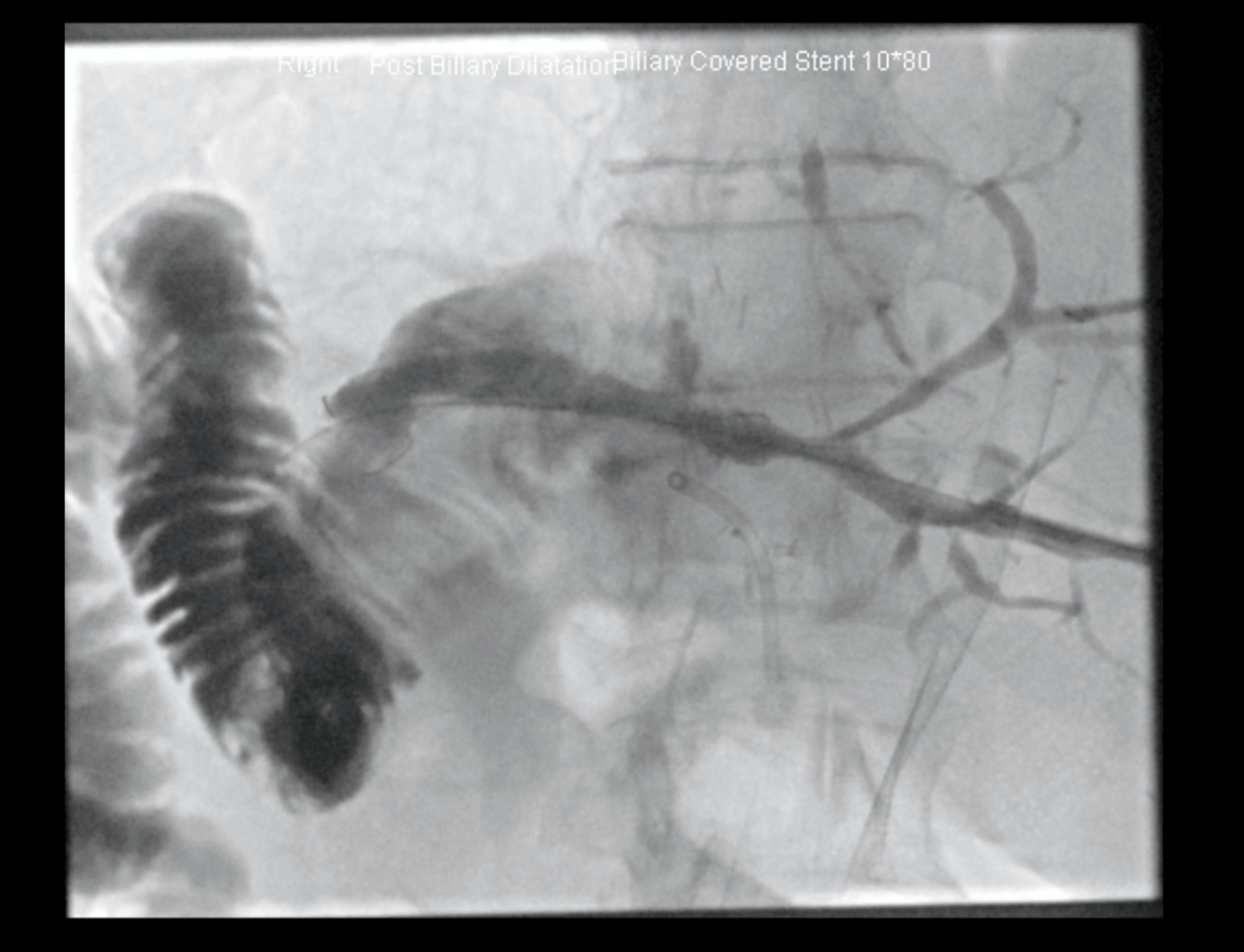
Novel biliary stent insertion via trans-jejunal approach Cholangiogram after placement of a metal stent confirmed satisfactory internal drainage into the Roux loop without any extravasation of contrast into the peritoneal cavity.

Roux loop catheterization was confirmed by injecting contrast (Omnipaque 300, GE Healthcare, USA). A guidewire (Storq 0.35, Cordis, USA) was then advanced into the Roux loop, and an 8 mm × 6 cm fully covered metal stent (Be Graft Peripheral, Bentley InnoMed GmbH, Germany) was deployed to recreate the biliary-enteric anastomosis (Figure 9).

**Figure 10:**
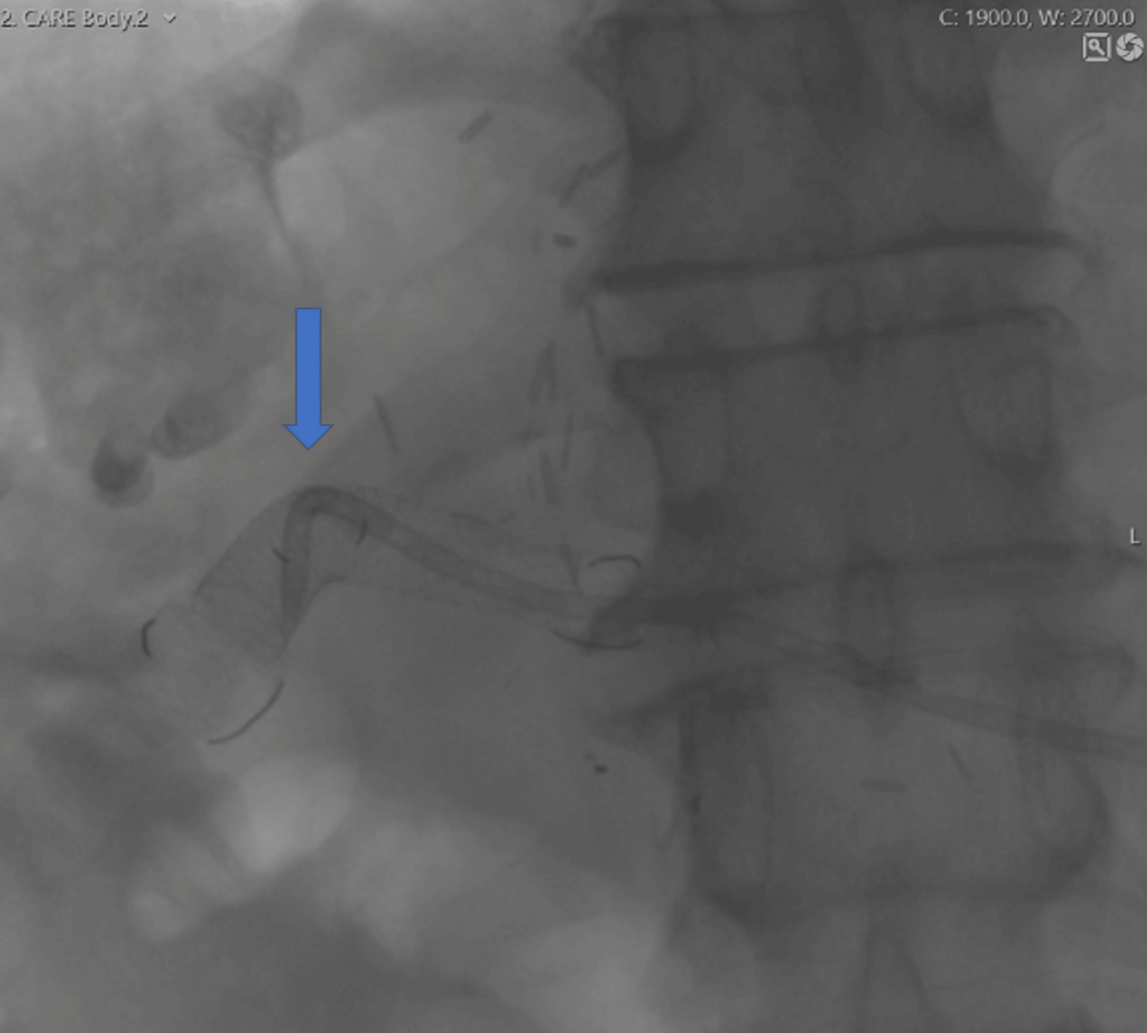
Novel biliary stent insertion via percutaneous trans-jejunal approach Successful deployment of the stent restoring bilio-enteric drainage (arrow). The PTC tube was kept as a guide for assessment. A tubogram was performed to ensure optimal stent position before removing the transhepatic catheter a few days before discharge. PTC: percutaneous cholangiogram

**Figure 11:**
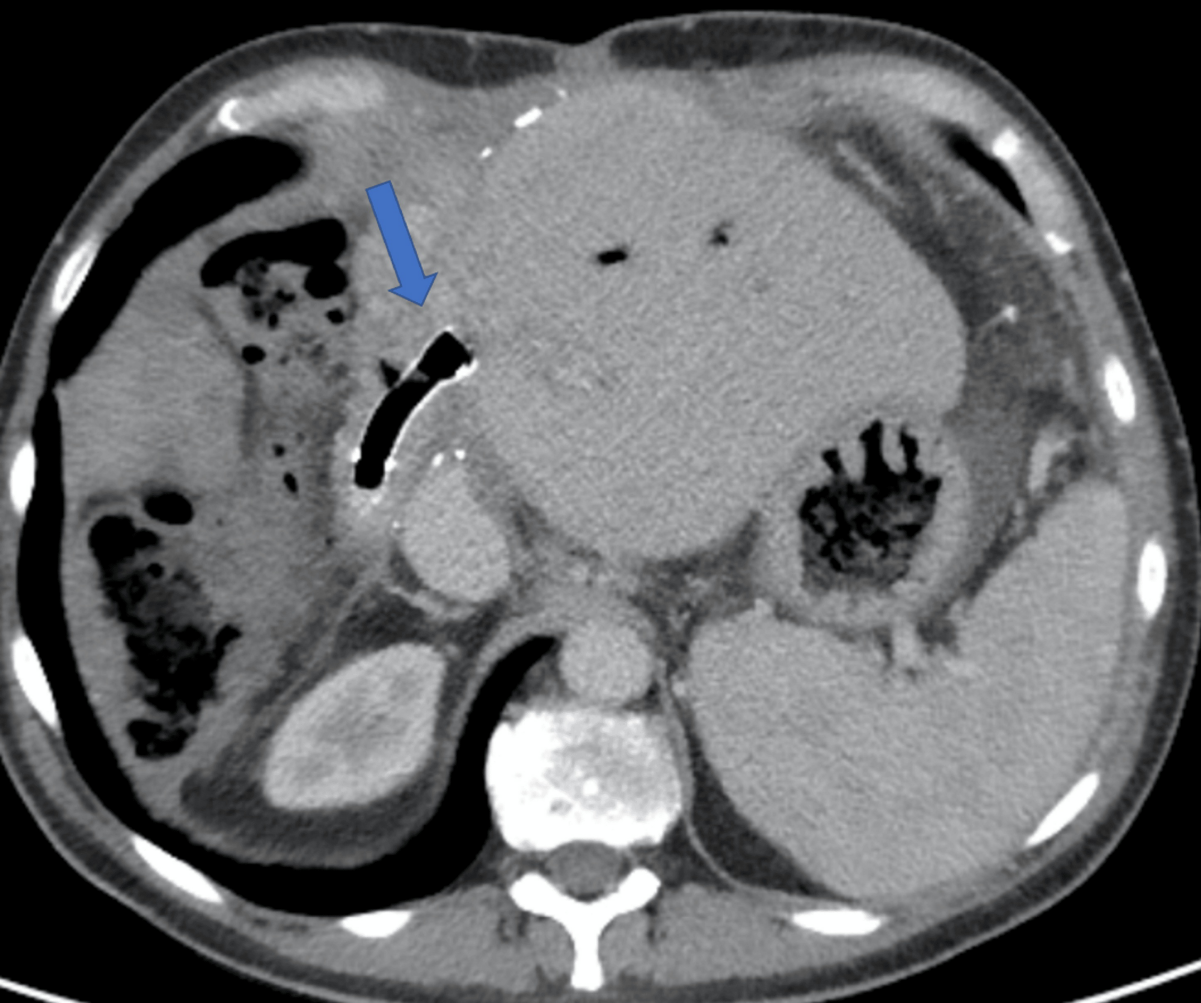
Novel biliary stent insertion via percutaneous trans-jejunal approach The satisfactory position of the metal stent (arrow) with CT demonstrates aerobilia and restored bilio-enteric drainage. CT: computed tomography

After the placement of the metal stent, a cholangiogram confirmed satisfactory internal drainage into the Roux loop, with no contrast extravasation into the peritoneal cavity. An external drain (Navarre Universal Drainage Catheter, BD, UK) was left in situ but was capped, and the patient was subsequently returned to the ward. A check tubogram was performed a week later, after which the external drain was removed. The patient was discharged two days post-procedure following a 98-day hospital admission (Figures 10-11).

The patient remained in good clinical condition at a follow-up appointment four weeks post-discharge, with satisfactory liver function test results. The only biochemical abnormality observed was mild cholestasis, characterized by a moderate increase in alkaline phosphatase and gamma-glutamyl transferase levels, while bilirubin remained normal. Repeat imaging demonstrated the resolution of intrahepatic biliary dilatation. The stent, which had facilitated bilio-enteric flow, had migrated to the small bowel without causing obstruction and was eventually passed in the stool without being detected. The portal vein appeared attenuated; however, Doppler ultrasonography confirmed good hepatopetal portal flow. The bilio-enteric anastomosis remained widely patent. As of 32 months post-surgery, the patient remains in good health, with no evidence of disease recurrence.

## Discussion

HCCA surgical management is one of the most challenging procedures in HPB surgical oncology. Curative resection with negative pathologic margins offers the best chance for long-term survival. The disease-specific five-year survival rate is reported to be 27% and can reach up to 58% in some series when en-bloc vascular resection is performed [1-3].

An ERH is often necessary to achieve optimal oncologic outcomes [2]. The complexity of this procedure arises from the intricate anatomical interrelations of structures affected by the tumor. Parenchymal resection, vascular reconstruction, and the restoration of anatomical continuity require a high level of expertise. Additional challenges may arise if postoperative complications occur. Moreover, the extent of liver resection necessitates preparatory volume manipulation to ensure an adequate FLR [2].

A hepatico-jejunostomy leak following HCCA resection has been reported in 9% to 20% of cases [3]. Several risk factors have been identified, including patient age, preoperative cholangitis, multiple anastomoses, and *Klebsiella* infection [6]. The biliary fistula in our case is classified as grade B, as it required active therapeutic intervention but was managed without relaparotomy. In contrast, grade C fistulas mandate relaparotomy [6]. Although classified as grade B, management proved particularly challenging due to its occurrence in the setting of concurrent liver dysfunction.

In such cases, a percutaneous transhepatic approach is preferred for treating an anastomotic stricture and re-establishing bilio-enteric communication. However, repeated failure of the traditional transhepatic approach complicated patient management. An alternative technique for establishing bilio-enteric continuity involves creating a gastro-hepatic fistula, accessing the biliary tree through a transluminal route under endoscopic ultrasound guidance. This approach has been reported in patients with bilio-enteric strictures following pancreatico-duodenectomy [7].

Kawasaki et al. described another endoscopic ultrasound-guided choledocho-jejunostomy using the saddle-cross technique, achieving internal fistulization through metal stent placement [8].

Yamaki et al. reported a double-balloon endoscopic retrograde cholangiography, another technique used for biliary strictures following pancreati-coduodenectomy. However, its failure rate is extremely high, up to 80%, when a complete anastomotic stricture is present [9].

Surgical intervention remains a last resort but carries significant risk in the postoperative setting, given the hostile environment created by post-biliary leaks and the hazardous nature of dissection through inflamed planes. This increases the risk of injury to major vascular structures. Historically, surgery in this setting has resulted in high mortality rates, reaching up to 77.8% [3].

The trans-jejunal approach for accessing the hepatico-jejunostomy is an old-fashioned technique. Traditionally, surgeons would fix the small bowel loop to the abdominal wall during planned bilio-enteric anastomosis, ensuring safe access to the hepatico-jejunostomy for potential future interventions [10].

This surgical maneuver, which involved transfixing the jejunal loop to the abdominal wall, was designed as a safety net for managing anastomotic complications such as strictures or bile leaks. However, this technique has become obsolete with advancements in interventional radiology and the widespread adoption of percutaneous transhepatic biliary access.

In our case, the trans-jejunal approach for catheterizing the Roux-en-Y loop using a TIPS needle was both innovative and successful. This technique was attempted after thoroughly discussing all other potential options deemed inapplicable or excessively hazardous.

## Conclusions

Studying the postoperative course of events, two scenarios were suspected to explain the radiological and clinical findings. In the first scenario, an evolving bile leak initially led to the formation of a complex collection, which exerted pressure on the anastomotic site, perpetuating the bile leak until it gradually emptied via a percutaneous route. However, the sustained pressure eventually resulted in anastomotic disruption. In the second scenario, an early postoperative hematoma underlying the anastomosis caused direct pressure on the anastomotic site, similarly leading to a bile leak and gradual disruption. Either of these mechanisms could explain the atypical progression of this case, in which a resolving anastomotic leak led to gradual anastomotic rupture. This rupture, in turn, was replaced by a lengthy, tight stricture, challenging the conventional management approaches for what is otherwise a well-managed complication.

This is the first case report of this type of interventional procedure used to treat a tight, lengthy bilio-enteric anastomosis following an ERH for HCCA. The intervention proved lifesaving, as the metal stent successfully established a durable bilio-enteric connection, which remained intact even after its early migration.
